# Shifting Baselines on a Tropical Forest Frontier: Extirpations Drive Declines in Local Ecological Knowledge

**DOI:** 10.1371/journal.pone.0086598

**Published:** 2014-01-21

**Authors:** Zhang Kai, Teoh Shu Woan, Li Jie, Eben Goodale, Kaoru Kitajima, Robert Bagchi, Rhett D. Harrison

**Affiliations:** 1 Program for Field Studies in Tropical Asia (www.pfs-tropasia.org), Xishuangbanna Tropical Botanical Garden, Chinese Academy of Sciences, Mengla, China; 2 Ecology, Conservation, and Environment Center (ECEC), State Key Laboratory of Genetic Resources and Evolution, Kunming Institute of Zoology, Chinese Academy of Sciences, Kunming, China; 3 Danau Girang Field Centre, Lower Kinabatangan Wildlife Sanctuary, Sandakan, Malaysia; 4 Bulong Nature Reserve, Jinghong, China; 5 Key Laboratory of Tropical Forest Ecology, Xishuangbanna Tropical Botanical Garden, Chinese Academy of Sciences, Mengla, China; 6 Department of Botany, University of Florida, Gainesville, United States of America; 7 Swiss Federal Institute of Technology (ETH) Zürich, Ecosystem Management Group, Institute of Terrestrial Ecosystems, Zürich, Switzerland; 8 Centre for Mountain Ecosystem Studies (CMES), Kunming Institute of Botany, Chinese Academy of Sciences, Kunming, China; 9 World Agroforestry Centre, East Asia Node, Kunming, China; Institut Pluridisciplinaire Hubert Curien, France

## Abstract

The value of local ecological knowledge (LEK) to conservation is increasingly recognised, but LEK is being rapidly lost as indigenous livelihoods change. Biodiversity loss is also a driver of the loss of LEK, but quantitative study is lacking. In our study landscape in SW China, a large proportion of species have been extirpated. Hence, we were interested to understand whether species extirpation might have led to an erosion of LEK and the implications this might have for conservation. So we investigated peoples' ability to name a selection of birds and mammals in their local language from pictures. Age was correlated to frequency of forest visits as a teenager and is likely to be closely correlated to other known drivers of the loss of LEK, such as declining forest dependence. We found men were better at identifying birds overall and that older people were better able to identify birds to the species as compared to group levels (approximately equivalent to genus). The effect of age was also stronger among women. However, after controlling for these factors, species abundance was by far the most important parameter in determining peoples' ability to name birds. People were unable to name any locally extirpated birds at the species level. However, contrary to expectations, people were better able to identify extirpated mammals at the species level than extant ones. However, extirpated mammals tend to be more charismatic species and several respondents indicated they were only familiar with them through TV documentaries. Younger people today cannot experience the sights and sounds of forest animals that their parents grew up with and, consequently, knowledge of these species is passing from cultural memory. We suggest that engaging older members of the community and linking the preservation of LEK to biodiversity conservation may help generate support for conservation.

## Introduction

Local ecological knowledge (LEK), which is synonymous with traditional ecological knowledge [1] or indigenous knowledge [2], can be defined as a cumulative body of knowledge and beliefs about the relationships of living beings (including humans) with one another and with their environment [3]. It is usually based on frequent observations at a restricted geographical scale and, hence, information about the taxonomy, life histories, behaviour, abundance, and habitat preferences of certain species, including for example preferred quarry or plants harvested for medicinal properties, can be very detailed (e.g. [4]–[7]). Moreover, LEK of sedentary communities that are tied to a specific resource base, such as a particular area of forest or wetland, is likely to be relevant to sustainable use of those resources and thus the conservation of biodiversity [2]. The level of LEK may also positive influence people's attitudes to conservation [8]. Consequently, the value of LEK to modern ecology and conservation is increasingly recognized [1], [8]–[11].

Rapid loss of LEK is a worldwide phenomenon as indigenous livelihoods change. Such loss may not only affect culture, but also reduce environmental awareness, diminish local capacity for sustainable resource use [12], [13], and negatively affect utilisation of biodiversity-based economic products (e.g. drugs [14]). The reasons for declining LEK are complex and multi-faceted [15]. Social factors are well documented, including migration (but see [16]), transition to market economies [17], [18], modern education [19], development related to rapid modernization and cultural homogenization [20], [21], and loss of access to traditional resources due to government intervention, including conservation programs [15]. At the same time, there are biological reasons for a decline in LEK. The biodiversity of tropical landscapes is being rapidly reduced and thus the subject of LEK is also being eroded [22]. Current theoretical constructs emphasize the parallel nature of the decline in biodiversity and cultural diversity [23]–[25], or “biocultural diversity” [26]. For example, once certain medicinal plants become rare, the knowledge and culture associated with these plants may also decline [27]. Greater quantitative study of the biological basis of LEK decline is therefore needed. In addition, not only is it possible that local knowledge of specific species and their attributes may be lost, but as people grow up in increasing altered environments perceived norms of the condition of local ecosystems may shift in the direction of the degraded environment. This process has been referred to as the shifting-baseline syndrome and can potentially undermine efforts to restore ecosystems, and the ecosystem services they provide, to their former condition [28], [29].

In this paper, we investigate the role of biodiversity loss in the loss of LEK in a tropical forest frontier region in Xishuangbanna, SW China. The region we studied is occupied by Akha people who have inhabited the montane areas of Xishuangbanna since the middle of the 18^th^ century and formerly practised swidden agriculture and hunter-gathering [30]. The long association between the Akha and their environment has led to the accumulation of a considerable body of LEK, which was formerly incorporated into village management systems to maintain the functional links between sustainable livelihoods, culture, and biodiversity [30], [31]. However, recent economic development and government policies have tended to sever these traditional ties with the land [32]–[34]. Today the study landscape still supports a relatively large area of natural forest, including a substantial area of montane rain forest, which is a highly threatened habitat. Moreover, forest area has increased and forest quality has improved since the nationwide logging ban in 1998. Recognising the potential importance of the area for biodiversity conservation, the perfectural government incorporated the natural forest area of the landscape into a nature reserve (35,000 ha) in 2009.

When the Akha first migrated into the area, hunting would have been integral to their livelihoods which depended to a large degree on forest produce. However, relatively low population densities and the use of traditional hunting technology presumably limited the impact on wildlife populations. In modern times, as has occurred throughout SE Asia [35]–[37], increased population, a switch to modern technology, including for example firearms and nylon mist nets, that require relatively little skill to use, and other factors, such as increased leisure time, have resulted in higher hunting pressure and the consequent extirpation of many species. Over 40% of the bird species and a substantial (but unknown) proportion of mammal species formerly occurring in the study landscape have been extirpated within the past 20–50 years (Sreekar et al, unpublished data). We were interested in understanding whether these extirpations may also have led to a decline in LEK and the possible implications this might have for conservation efforts.

To assess peoples' LEK, we investigated their ability to name local species in the local language. Clearly, LEK is more complex and nuanced than an ability to name species. Nevertheless, names are an essential component of any system of LEK [38], as the labels that link information about the attributes of species and their environment. In the absence of ability to name species cultural transmission of LEK would be impossible (or at least extremely limited). Moreover, many indigenous peoples, including the Akha, employ a hierarchical system of naming [39], [40] (but see [41]), which implicitly identifies relationships among species. In addition, this property of the naming system enables a semi-quantitative assessment of a person's familiarity with a particular species by assessing whether they can name the species at the specific level, the generic level, or not at all. Finally, testing peoples' ability to name species can be done relatively easily in an unbiased and repeatable manner, thus is a widely used method to study LEK (e.g. [12], [42], [43]).

To assess the possible impact of biodiversity loss on LEK in the study landscape, we investigated the hypothesis that people were less able to identify extirpated species than extant species. Clearly, it may be expected that on average people would be less able to identify rare species than common species, simply because they encounter them less frequently. However, whereas rarity may reflect low natural abundance or biodiversity loss (or both), extirpated species by definition have declined in abundance. Therefore, a difference in the ability to identify extirpated species compared with extant species indicates an impact of biodiversity loss. We structured sampling so that we were able to examine and control for the effects of gender and age, which is likely to covary with other drivers of LEK loss, such as formal education and economic development.

## Methods

We conducted our research in and around Bulong Nature Reserve in Mengsong township (UTM/WGS84: 47N 656355 E, 2377646 N), Xishuangbanna, China. Mengsong varies from 800 m to 1800 m asl and has a tropical climate influenced by the Indian monsoon (mean annual temperature = 18°C at 1600 m asl and annual rainfall = 1600–1800 mm; [44]). The principle vegetation types are montane rain forest and evergreen broadleaf forest [44].

According to a 2010 census, there were 2024 people living in six Akha villages in southern Mengsong, which we took as our sample population. We short-listed people who fulfilled the following four criteria: (1) aged between 20 and 60 years, (2) born and (3) currently living in one of the six selected hamlets, and (4) in a healthy mental and physical state. We did not want to interview people younger than 20 years, since answers might reflect developmental differences, and the population of people older than 60 who were in a fit physical and mental state was small. Criteria (2) and (3) were included to bound our sample population to those that had grown up and still lived in the study landscape. We wished to limit our sample to those in a fit physical and mental state, as disabilities might affect a person's ability to visit the forest or to give reliable answers. Population information was obtained through interviews with village headmen and from local government records. People were then classified into four categories according to age and gender, and we randomly selected 30 villagers from each category. When people were unavailable, we randomly re-selected replacements from the original list. We were finally able to complete 113 interviews (30 males 20–40 yrs; 30 males 41–60 yrs; 26 females 20–40 yrs; and 27 females 41–60 yrs).

ZK collected Akha names of bird species during an exhaustive bird survey of the area in 2010–2012. These names were obtained from two principle key informants, who were assisting with the bird survey. We classified birds to three abundance categories based on these earlier surveys; a) common, b) rare and c) locally extirpated species, and randomly selected ten species from each category. We identified locally extirpated species by comparing our bird checklist with an earlier bird checklist compiled by biologists between 1994 and 2000 [45] and an inferred checklist generated from a list of all species expected to occur in the area based on range, habitat preference and elevation distribution from [46], [47] (Table S1, Sreekar et al, unpublished data). We also included five bird species in our questionnaire that have never occurred in the region, but that have congeneric species that do, as a quality control. We repeated one species in the questionnaire to check interviewees’ consistency. Thus, the final list comprised 36 bird species (online supplementary material, Table S1).

Data on mammals were more limited. We compiled a list of mammals of the area through group discussions with six key informants, all of whom were experienced hunters. We showed the group colour drawings of mammals and asked them to name species they had encountered in the field. Then, based on the group's advice, we classified these mammals into two categories; a) extant and b) extirpated. Five species were randomly selected in each category. However, one species initially classified as extirpated turned out to be extant but very rare. Thus, we ended up with six extant species and four extirpated species in the sample. We also included three species that have never occurred in the region, but that have congeneric species that do, and one repeated species as quality controls (online supplementary material, Table S1). Note that none of the key informants were selected for interview. For all selected species, we prepared pictures for identification using [48] and [49] as sources.

All interviews were conducted individually in November 2012. One of our group members (LJ) is a local Akha, and his presence during the interviews allowed us to conduct most of them in Akha. The interviewees were required to identify the birds and mammals using Akha names, which are binomial with the group-level name being roughly equivalent to genus (Table S1). Identifications were scored as incorrect, correct at the group-level, or correct at the species-level. When people answered with the specific name of a congeneric species, we accepted this as being correct at the group-level.

For each species, two further questions were asked: (1) If they had seen the species before and (2) if they still see it now. Interviewees who gave a positive answer to the first and a negative answer to the second question were asked a further question; (3) when was the last time they saw the species. Answers to these questions were only used for respondents who gave a correct identification at the species level. A further two open ended questions on forest usage and the animals that they thought had been extirpated from Mengsong were asked. (1) How often did you go to the forest when you were a teenager (ordered factor: 1, less than once per month; 2, ≥ once per month but ≤ once per week; 3, more than once per week)? (2) What animals do you remember were present in the past but are absent now?

We analysed the data for birds and mammals separately. We modeled the frequency of answers in each identification category (no identification, group level identification, and species level identification) using a Poisson (link = log) generalised linear mixed model (package *lme4*, function *glmer*; [50]). Full model details and results are given in the online supplementary materials (Table S2 and Table S3). We included identification-level as a fixed effect and used *a priori* contrasts to compare between (i) species not identified and species identified to either group or species levels, and (ii) between species identified to group level and species level. Hence, to measure the effect of the explanatory variables (respondent gender, respondent age, species abundance, and their two-way interactions) on respondents’ ability to identify species, we considered the interactions between identification level and these explanatory variables [51]. For example, if the coefficient for the id_level:age term for the species versus group level comparison was positive and significant, it would indicate that older people were more likely to have named animals correctly at the specific level. As we were interested in the effect of species extirpation on LEK, our factor of primary interest was species abundance. Gender was included as a covariate, because traditional divisions of labor are likely to result in differences in LEK among sexes [52]. Meanwhile, age was considered as many studies have shown that LEK bears a positive association with age [53], [54]. First, older people will have had, on average, more opportunity to accumulate LEK. Second, in an area of rapid development, age is likely to be closely correlated with other known drivers of the loss of LEK, including exposure to modern schooling, degree of forest dependence, and changes in language usage. Thus, by including age as a covariate, we hope to have controlled for these other development related drivers that we were not able to study explicitly. To account for non-independence of answers from the same individual and individuals from the same village, both village and individual nested within village were included in the model as normally-distributed random effects. We analysed the association between frequency of forest visits as a teenager and age using Spearman Rank correlation. All analyses were carried out in R [55].

An extirpation time-line for five species of mammals in Mengsong was constructed from the responses of knowledgeable interviewees – those who correctly identified the quality control species.

Government authorities in Mengsong agreed to participate in the study and provided demographic information. Selected interviewees were approached and asked if they wish to participate. Anyone that did not wish to participate (3 people) was not interviewed. Only verbal consent was obtained. Our questionnaire and interview methodology comply with the Society for Ethnobiology and Yunnan Initiative guidelines for social surveys and we received approval from the Kunming Institute of Botany ethics committee to conduct the work.

All data generated through this study and the code used in the analysis may be accessed and downloaded at www.datadryad.org (doi:10.5061/dryad.5j56v).

## Results

The respondents' stated frequency of forest visits as a teenager was correlated with age for men (Sperman rank correlation, *ρ* = 0.32, n = 1252, *p* = 0.0265) and marginally significantly correlated for women (Sperman rank correlation, *ρ = *0.25, n = 1573, *p* = 0.0869). Thus, younger people and especially younger men, visited the forest less frequently as teenagers than older people had done.

**Figure 1:**
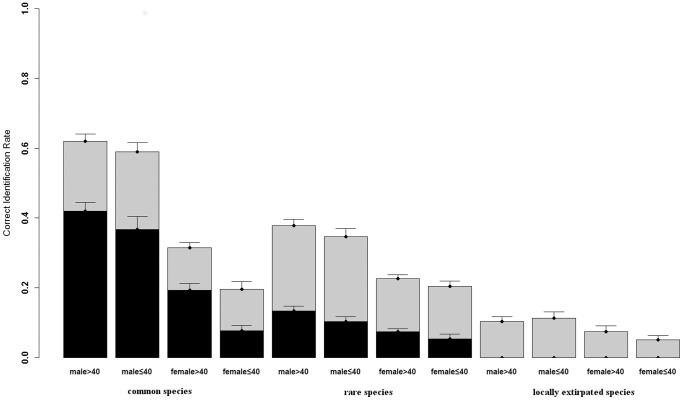
Proportion of bird species identified to group level (light grey) or identified to species level (dark grey) against respondent gender, respondent age, and species abundance (error bars = standard error). Note that for the statistical modeling respondent age was treated as a continuous variable.

For birds, only 15% of responses were correct at the group-level and 12% at the species-level, and no one in our sample was able to name a locally extirpated species at the species-level (Figure 1). The model selection process and parameter coefficients for the best model to explain peoples' ability to identify birds are summarised in Tables 1 and 2, respectively. Full model details are presented in the online supplementary material (Table S2). Compared with gender and age, species abundance had by far the largest effect on peoples' ability to identify birds (Figure 1, Table 2). People were more likely to identify common species than rare species and rare species than locally extirpated species (Table 2). In addition, women identified fewer birds overall, but there was no significant gender effect on the ability to identify birds at the species level as compared to the group level (Table 2). Age had no effect on the ability to identify birds overall, but there was a significant positive effect of age on the ability to name birds at the species as compared to the group level (Table 2). There was also a gender-age interaction: The age effect was significantly greater in women (Table 2).

**Table 1:** Summary of the selection process for the model for bird identification.

Model parameters included	K	ΔAIC
null model	9	730
gender + age + abundance	17	2.7
gender + age + abundance + gender:age	20	0
gender + age + abundance + age:abundance	23	8

* *We could not examine the gender:abundance interaction because of complications with the Hauck-Donner effect.*

We modeled the frequency of identifications at a particular level using a Poisson (link = log) GLMM, with village and respondent nested within village included as random effects (not shown). We investigated the effect of respondent gender, respondent age, and species abundance (common, rare, or extirpated) and their interactive effects* on the ability of people to correctly name species at two levels (overall (group+species level) and specific (species vs group level)). Full model details are given in the online supplementary material Table S2. Starting with the null model, we added and subtracted parameters by hand and assessed the impact of a factor by comparing AIC values. K = number of model parameters. ΔAIC_c_ = difference between AIC_c_ of the top ranked model and current model.

**Table 2:** Summary of the parameter coefficients for the best model for bird identification.

Parameter	β coefficient	Std. Error	z value	Pr(>|z|)
overall: gender	−0.634	0.111	−5.70	<0.0001
specific: gender	−0.377	0.297	−1.27	0.2037
overall: age	−0.000	0.002	0.164	0.8700
specific: age	0.008	0.004	2.245	0.0248
overall: species abundance(1)	−0.871	0.120	−7.29	<0.0001
specific: species abundance (1)	−1.786	0.357	−5.01	<0.0001
overall: species abundance (2)	−0.315	0.072	−4.41	<0.0001
specific: species abundance (2)	−0.523	0.212	−2.47	0.0134
overall: gender:age	0.015	0.009	−1.80	0.0716
specific: gender:age	0.023	0.011	2.12	0.0341

We used a binomial (link = logit) GLMM with village and respondent nested within village included as random effects (not shown). We investigated the effect of respondent gender, respondent age, and species abundance ((1) common vs rare, (2) rare vs extirpated) and their interactive effects on the ability of people to correctly names species at two levels (overall (group+species levels) and specific (species vs groups levels)). Full model details are given in the online supplementary material Table S2.

**Figure 2:**
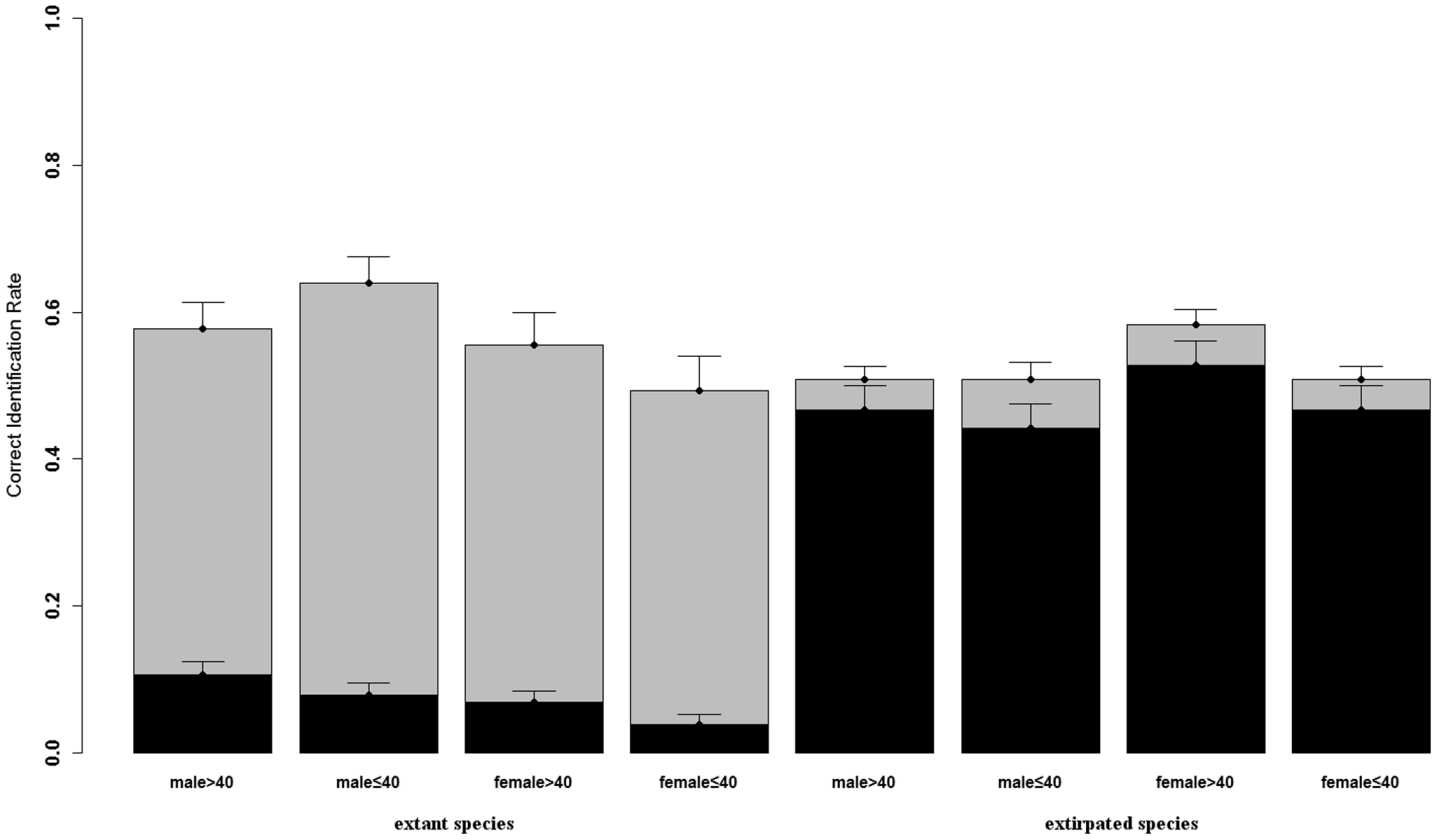
Proportion of mammal species identified to group level (light grey) or identified to species level (dark grey) against respondent gender, respondent age, and species abundance (error bars = standard error). Note that for the statistical modeling respondent age was treated as a continuous variable.

For mammals, 32% of responses were correct at the group-level and 23% at the species-level (Figure 2). The model selection process and parameter coefficients for the best model to explain peoples' ability to identify mammals are summarised in Tables 3 and 4, respectively. Full model details are presented in the online supplementary material (Table S3). Again, species abundance was the most important factor in determining peoples' ability to name species (Table 4). People were poorer at identifying extirpated species overall (Table 4). However, contrary to expectations, people were able to name locally extirpated species better than extant mammals at the species as compared to the group levels (Table 4). Several respondents indicated that they were familiar with extirpated mammals because they had seen TV documentaries about them. There was no significant effect of gender or age on the ability to identify mammals at either the overall or the specific level (Table 4), although the age effect was marginally significant (P = 0.075) at the species level. Moreover, none of the interactive effects were significant and were removed during model selection (Table 3).

**Table 3:** Summary of the selection process for the model for mammal identification.

Model parameters included	K	ΔAIC
null model	9	401
gender + age + abundance	15	0
gender + age + abundance + gender:age	18	2
gender + age+ abundance + age:abundance	18	4.4
gender + age + abundance + gender:abundance	18	0.8

We modeled the frequency of identifications at a particular level using a Poisson (link = log) GLMM, with village and respondent nested within village included as random effects (not shown). We investigated the effect of respondent gender, respondent age, and species abundance (extant or extirpated) and their interactive effects on the ability of people to correctly name species at two levels (overall (group+species level) and specific (species vs group levels)). Full model details are given in the online supplementary material Table S3. Starting with the null model, we added and subtracted parameters by hand and assessed the impact of a factor by comparing AIC values. K = number of model parameters. ΔAIC_c_ = difference between AIC_c_ of the top ranked model and current model.

**Table 4:** Summary of the parameter coefficients for the best model for mammal identification.

Parameter	β coefficient	Std. Error	z value	Pr(>|z|)
overall: gender	−0.062	0.040	−1.55	0.1206
specific: gender	−0.002	0.082	−0.03	0.9770
overall: age	0.002	0.002	1.30	0.1924
specific: age	0.007	0.004	1.78	0.0749
overall:abundance	−0.092	0.039	−2.38	0.0172
specific:abundance	1.484	0.097	15.34	<0.0001

We modeled the frequency of identifications at a particular level using a Poisson (link = log) GLMM, with village and respondent nested within village included as random effects (not shown). We investigated the effect of respondent gender, respondent age, and species abundance (extant vs extirpated) and their interactive effects on the ability of people to correctly names species at two levels (overall (group + species levels) and specific (species vs groups levels)). The interactive terms were removed during model simplification. Although models including the interactive terms were roughly equivalent (Table 3), none of the coefficients for the interactive terms were significant. Full model details are given in the online supplementary material Table S3.

**Figure 3:**
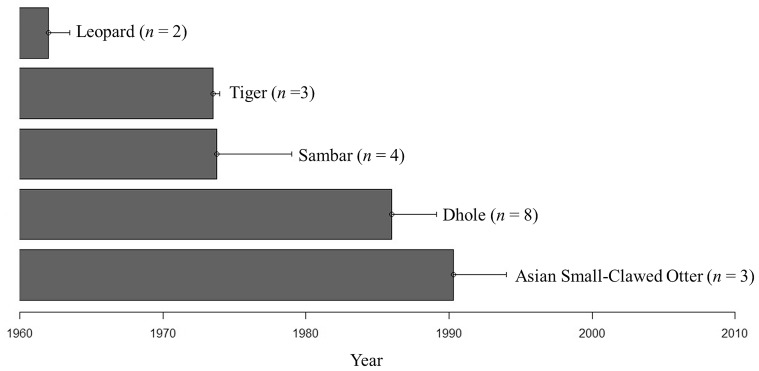
A local extirpation time-line for five mammal species in Mengsong, SW Xishuangbanna, China (*n* refers to the number of respondents who provided time-line data for the species). Only answers from respondents who identified the species concerned and who correctly identified the quality control species (see Methods) were used in constructing the extirpation time-line. In total 113 people were interviewed.

We were able to establish an extirpation time line for five mammal species: Asian small-clawed otter (*Aonyx cinerea*), Dhole (*Cuon alpinus*), Sambar (*Rusa unicolor*), Leopard (*Panthera pardus*), and Tiger (*Panthera tigris*). These local extirpations were traced to between 1960s and 1990s (Figure 3).

## Discussion

Our study suggests a rapid erosion of LEK on a tropical forest frontier in SW China. Based on our extirpation time-line, most mammals appear to have been extirpated from the landscape within the past 40 yrs and at least 37 bird species were extirpated within the past 20 yrs (Sreekar et al, unpublished data). No one in our sample was able to name a locally extirpated bird at the species level and just 9.5% of responses were correct at the group-level. For mammals the overall percentage of correct answers was higher (55%). For both birds and mammals, species abundance had a far larger effect on peoples' ability to name species than either gender or age. People were much less likely to identify extirpated birds than common or rare species – at either the group or species level. This suggests a substantial impact of biodiversity loss on LEK. It is worth noting that some of the locally extirpated birds were distinctive species that would normally have been common in the absence of hunting (Table S1). People were also more likely to name extant mammals than extirpated ones overall. However, contrary to expectations, people named more extirpated mammals to the species-level, although this appears to reflect modern knowledge derived from TV rather than familiarity with the species in their natural setting.

A few caveats are warranted here. First, we used peoples' ability to identify species as an index of LEK. As we have argued, we believe this is a useful index because species names are an essential component of any system of LEK and a person's ability to identify species can be easily surveyed in a quantifiable and repeatable manner. Nevertheless, the exact relationship between an ability to name species and other components of LEK is not known [43], [56]. Second, as discussed earlier, age is likely to be closely correlated with known socioeconomic drivers of declines in LEK, such as involvement in formal education or the market economy. Thus, by controlling the effects of age in our models, we hope to have accounted for the effects of these other drivers. However, we were not able to examine these other drivers explicitly. Ultimately, this comes down to the problem that this was, by necessity, a correlative study and, therefore, causation cannot be assigned unambiguously. Perhaps more importantly, none of these factors are operating in isolation and, although the independent effects may be measured statistically, it is their integrated impact that determines LEK loss. Finally, it should be acknowledged that people may be more familiar with the calls or habits of some animals [4], [57] and, hence, using peoples' ability to identify pictures may under-estimate their knowledge of these species. However, with a random selection of species, as we employed, this is unlikely to bias the results in any particular direction.

Traditional divisions of labor in indigenous communities result in gender differences in knowledge about their environment. For example, men are often better able to identify game animals, because of their traditional involvement in hunting [52]. We found that men were better able to identify birds overall, but there was no difference between genders in their ability to name birds at the species as compared to group levels. Interestingly, there was no effect of gender on peoples' ability to name mammals.

Age did not have a significant effect on peoples' ability to name birds overall, but older people were better able to name birds at the species as compared to the group level. This suggests an erosion of LEK through time and is consistent with the fact that younger people visited the forest less frequently as a teenager. Presumably, this is in part because of modern schooling, and may also reflect the greater involvement of younger people in the market economy and other development related changes [52]. The effect of age was also stronger in women than in men. This may possibly reflect the fact that, although younger people are less dependent on the forest, young men still like to go hunting. By comparison, most of the young women we interviewed professed to only rarely visiting the forest.

People were more likely to identify extirpated mammals than extant ones at the species level. This result may reflect a taxonomic bias, as most of the extirpated mammals were the more charismatic carnivores, such as Tiger and Dhole. Several of the respondents also informed us that they were familiar with particular species through TV documentaries, which obviously tend to focus on more charismatic animals. This possibly also explains why neither gender nor age had a significant effect on peoples' ability to name mammals. Nevertheless, this observation suggests that, even in a forest frontier region, modern media may be an effective way to enhance peoples' knowledge of their environment in the face of declining traditional LEK.

In Mengsong young people today cannot experience the sights and sounds of the forest their parents grew up with and consequently knowledge of many local species is being lost from cultural memory. A large proportion (60%) of respondents admitted to never having seen a Silver Pheasant (*Lophura nycthemera*), although this species is an important cultural emblem among Akha. A similar process is occurring throughout much of the tropics [58]. For example, in Sarawak depictions of the Rhinoceros Hornbill (*Buceros rhinoceros*) in local art are abundant and the state is known as the “Land of the Hornbill” but, following its extirpation from all but the remotest forests, few local people ever have the opportunity of seeing this impressive bird. One of the main drivers of the extirpation of *Buceros* hornbills has been the use of their tail feathers in the costumes of local dance troops who perform for tourists and government officials [59].

As is often the case in tropical forests [58], in our study landscape intense local hunting most likely accounts for the extirpation of most birds and mammals. Two lines of evidence support this notion. First, encounter rates with hunters carrying guns (i.e. not including other forms of hunting) is high. On average, over a two year period we passed a hunter or a hunting party every two hours when walking on trails (Sreekar et al., unpublished data). Second, the forest area has increased and forest quality improved over the past 15 years, suggesting area effects and habitat quality effects are unlikely causes of species extirpations. It is worth noting that modern hunting in Mengsong is very different from its traditional progenitor, not only in the use of modern firearms and other technology (e.g. motorcycles for access), but also in that hunting is now essentially a sport. The rarity of successful hunts and diminishing size of quarry means that bush-meat can no longer represent an important source of nutrition or income for local people. One interviewee reported that twenty years previously he could hunt two civets a night, but that it now takes him two weeks or longer to find one. Yet clearly this did not deter his interest in hunting. It is also clear that people still like to eat wild meat, although local resources have been largely exhausted. It is not uncommon to see people barbecuing tiny birds, which they have caught using mist-nets, or buying bush-meat from across the border in Myanmar.

Rapid erosion of LEK, combined with biodiversity loss as one of the main drivers, indicates a potential for positive feedbacks that could make future conservation efforts increasingly difficult [29]. Successful conservation throughout SE Asia [37], [60] and elsewhere in the tropics [61] will require reducing hunting pressure to sustainable levels. Although, stricter law enforcement is important, it is also essential to cultivate a conservation ethic. We suggest this may be achieved through linking biodiversity conservation to the preservation of indigenous culture - specifically the stories, ceremonies, and practical uses associated with rare and extirpated species. In Mengsong, there are still a few older people who retain a large amount of LEK. We suggest education programs about the local environment could incorporate these older community members' experiences of the forest.

## Supporting Information

Table S1 **List of species used in the questionnaire.**
(DOC)

Table S2 **Summary table of the best model for bird identification.**
(DOC)

Table S3 **Summary table of the best model for mammal identification.**
(DOC)
