# Correction: Shifting Baselines on a Tropical Forest Frontier: Extirpations Drive Declines in Local Ecological Knowledge

**DOI:** 10.1371/journal.pone.0092931

**Published:** 2014-03-12

**Authors:** The PLOS ONE Staff

There was an error in the Funding statement. The Funding statement should read: “Funding for AFEC-X 2012 was provided by Xishuangbanna Tropical Botanical Garden (XTBG), World Agroforestry Institute (ICRAF, East Asia node) Kunming Office, and the Key Laboratory for Tropical Forest Ecology (XTBG). The funders had no role in study design, data collection and analysis, decision to publish, or preparation of the manuscript.” The publisher apologizes for this error.
