# Supplementary material for: Shifting Baselines on a Tropical Forest Frontier: Extirpations Drive Declines in Local Ecological Knowledge
Source: PLoS One. 2014 Jan 21;9(1):e86598. doi: 10.1371/journal.pone.0086598 (PMC3897741; doi:10.1371/journal.pone.0086598)
Supplement: Table S1 — List of species used in the questionnaire. (DOC) [file pone.0086598.s001.doc]

**Table S1** Bird and mammal species used in the questionnaires. The order species are listed here is systematic, but the order species were listed in the questionnaire was random. Akha is not normally written and the transcribing of animals names here was according to the Chinese government's official phonetic rendition by one (LJ) of us who is a native Akha speaker. We assigned Akha names at two levels; (i) species names and (ii) group names for assemblages of closely related species. If, when asked to name a picture, respondents gave the name of a congeneric species we assigned this as being correct at the group-level. Some group-level names are missing, because for very distinctive species sometimes only a specific Akha name applies. We could not obtain species-level names for all species, in particular some of the locally extirpated species. Respondents usually professed to not knowing these species or gave the group-level name. In a few cases, respondents gave obviously incorrect names, usually of other well-known species, and hence these answers could be scored as incorrect without difficulty. The abundance of birds was estimated from surveys conducted in 2010-2012 (unpublished data). In that study, locally extirpated birds were identified using two methods. First, recent extirpations were identified by comparing lists of species observed by [45] between 1994 and 2000 with the recent observations. Older inferred extirpations were estimated by generating a list of all species expected to occur in the area based on range, habitat preference and elevation distribution from [46,47]. Information about mammals was obtained from group discussions with key informants (experienced hunters), who were not selected for interview.

| **English name** | **Akha group name** | **Akha species name** | **Abundance** |
| --- | --- | --- | --- |
| ***Birds*** |  |  |  |
| Japanese Quail (*Coturnix japonica*) | Khanq zaq lavq ma |  | locally inferred extirpated |
| Mountain Bamboo Partridge (*Bambusicola fytchii*) | Gha caer | Gha yev | common |
| Silver Pheasant (*Lophura nycthemera*) |  | Gevq | rare |
| Striated Heron (*Butorides striata*) | Eer dzoeq |  | locally inferred extirpated |
| Black-winged Kite (*Elanus caeruleus*) | Haq dzeir |  | common |
| Northern Lapwing (*Vanellus vanellus*) |  |  | locally inferred extirpated |
| Speckled Wood Pigeon (*Columba hodgsonii*) | Khaq guq | Khaq guq nav | rare |
| Mountain Imperial Pigeon (*Ducula badia*) | Khaq guq | Khaq guq hanq | rare |
| Eastern Grass Owl (*Tyto longimembris*) | hawq bu aqma | Ar nyovq myav khanq | rare |
| Red-headed Trogon (*Harpactes erythrocephalus*) |  | Nga dzawq | locally extirpated |
| Great Hornbill (*Buceros bicornis*) | - | - | absent |
| Great Barbet (*Megalaima virens*) | Aq covr lov | Cur lu | common |
| Scarlet Minivet (*Pericrocotus flammeus*) |  | Aq jaq lavq pyawv | common |
| Bull-headed Shrike (*Lanius bucephalus*) | - | - | absent |
| Ashy Drongo (*Dicrurus leucophaeus*) | Jir joe |  | rare |
| Hair-crested Drongo (*Dicrurus hottentottus*) | Jir joe | Jir joe awva-avq | common |
| Red-billed Blue Magpie (*Urocissa erythrorhyncha*) |  | Tanq caeq | common |
| White-winged Magpie (*Urocissa whiteheadi*) | Tanq caeq |  | locally inferred extirpated |
| Common Magpie (*Pica pica*) | Tanq caeq |  | locally inferred extirpated |
| Black-crested Bulbul (*Pycnonotus flaviventris*) | Man yaevq | Man yaevq xeer | common |
| Black Bulbul (*Hypsipetes leucocephalus*) |  | Sev yaev | common |
| Rufous-faced Warbler (*Abroscopus albogularis*) | Cov sir |  | locally extirpated |
| Manchurian Bush Warbler (*Cettia canturians*) | Cov sir |  | locally extirpated |
| La Touche's Leaf Warbler (*Phylloscopus claudiae*) | Cov sir |  | common |
| Striated Prinia (*Prinia crinigera*) | - | - | absent |
| Rufous-vented Laughingthrush (*Dryonastes gularis*) | - | - | absent |
| Streak-throated Fulvetta (*Fulvetta manipurensis*) | Khaq bovq aq xaw / Khaq zaq myav xaw |  | rare |
| Asian Fairy Bluebird (*Irena puella*) |  |  | locally extirpated |
| Beautiful Nuthatch (*Sitta formosa*) | - | - | absent |
| Oriental Magpie Robin (*Copsychus saularis*) |  | Anr biq ar jir | rare |
| White-tailed Robin (*Myiomela leucura*) |  |  | rare |
| Blue Rock Thrush (*Monticola solitarius*) | Jaevq-awvq |  | rare |
| Ferruginous Flycatcher (*Muscicapa ferruginea*) | Khaeq awvq |  | rare |
| Ultramarine Flycatcher (*Ficedula superciliaris*) | Khaeq awvq |  | locally extirpated |
| Little Bunting (*Emberiza pusilla*) | Jar tsev | Jar tseevq | common |
|  |  |  |  |
| ***Mammals*** |  |  |  |
| Eurasian Red Squirrel (*Sciurus vulgaris*) | - | - | absent |
| Indian Giant Flying Squirrel (*Petaurista philippensis*) | ho xuq / ho byaw | ho xuq peer | extant |
| Pallas's Squirrel (*Callosciurus erythraeus*) | ho baq | ho danr | extant |
| Lesser Bamboo Rat (*Cannomys badius*) | ho piq | ho piq baq peer | extant |
| Yunnan Hare (*Lepus comus*) | lanq | lanq xeer | extant |
| Tiger (*Panthera tigris*) | khaq zeeq | khaq laq | extirpated |
| Binturong (*Arctictis binturong*) | pyaq ivq | pyaq ivq nav | extirpated |
| Small Indian Civet (*Viverricula indica*) | pyaq ivq | pyaq ivq byavq | extant |
| Dhole (*Cuon alpinus*) |  | khaq yaer | extirpated |
| Raccoon Dog (*Nyctereutes procyonoides*) | - | - | absent |
| Sun Bear (*Helarctos malayanus*) | khaq hmr | khaq hmr cir | extant |
| Asian Small-Clawed Otter (*Aonyx cinerea*) |  | eer xmr | extirpated |
| White-Lipped Deer (*Przewalskium albirostris*) | - | - | absent |
