# Supplementary material for: Shifting Baselines on a Tropical Forest Frontier: Extirpations Drive Declines in Local Ecological Knowledge
Source: PLoS One. 2014 Jan 21;9(1):e86598. doi: 10.1371/journal.pone.0086598 (PMC3897741; doi:10.1371/journal.pone.0086598)
Supplement: Table S2 — Summary table of the best model for bird identification. (DOC) [file pone.0086598.s002.doc]

**Table S2.** Detailed results of the best model for bird identification. The ability to identify a species at a particular level was modeled as a multinomial response in a surrogate Poisson (link=log) model. Thus the parameters of interest (highlighted in bold) are the interactions between id_level and the explanatory variables (respondent age (continuous), respondent gender (gender: male=0, female=1), species abundance (ordered factor: common, rare, locally extirpated), and their two-way interactions [51]. Note that the main effects are marginal to the effects of interest and hence cannot be removed from the model. We used *a priori* contrasts for abundance and iden_level to compare (1) common vs rare and (2) rare vs locally extirpated, and to compare (1) no id vs group + species level id (overall), and (2) group vs species level id (specific), respectively. Village and individual respondent identity (ID) nested within village were treated as a random effects. We used AIC to select the most appropriate model and parameters were added and removed by hand in a stepwise manner. We considered all two way interactions between age, gender and species abundance (with identification level), but not three-way interactions. The only parameters not included in the best model was the abundance:gender interactions. The ∆AIC of the second best model was 2.7. The model was slightly under-dispersed (∑Pearson residuals^2 = 881, residual df = 1001).

In addition, because no one was able to identify a locally extirpated bird at the species level, we had problems with the Hauck-Donner effect [51]. Hence, we selected one young women (age = 30), to be conservative with respect to the direction of the effects, and changed the identification level of one correctly identified locally extirpated species from the group to the species level. This had minimal impact on the estimates of other factors but enabled us to estimate the probability for the species abundance effect at the species-level. However, because of the influence of this point we were not able to investigate the gender:abundance interaction term.

| Generalized linear mixed model fit by maximum likelihood ['glmerMod'] | | | | |
| --- | --- | --- | --- | --- |
| Family: poisson ( log ) | | | | |
| Formula: yes ~ iden_level + gender + age + abundance + (1 | hamlet) + (1 | hamlet:ID) + iden_level:gender + iden_level:age + iden_level:abundance + iden_level:gender:age  Data: birds  AIC BIC logLik deviance  3043.245 3141.738 -1501.623 3003.245  Random effects:  Groups Name Variance Std.Dev.  hamlet:ID (Intercept) 2.889e-13 5.375e-07  hamlet (Intercept) 2.215e-13 4.706e-07  Number of obs: 1017, groups: hamlet:ID, 113; hamlet, 6 | | | | |
| Fixed effects | Estimate | Std. Error | z value | Pr(>|z|) |
| (Intercept) | 0.4764639 | 0.1542284 | 3.089 | 0.00201 ** |
| iden_level-overall | -0.6843274 | 0.0836319 | -8.183 | 2.78e-16 *** |
| iden_level-specific | -1.0770811 | 0.2244467 | -4.799 | 1.60e-06 *** |
| gender | -0.8871274 | 0.2042647 | -4.343 | 1.41e-05 *** |
| age | 0.0003167 | 0.0026134 | 0.121 | 0.90354 |
| abundance(1) | -1.3943472 | 0.2381995 | -5.854 | 4.81e-09 *** |
| abundance(2) | -0.6218389 | 0.1415594 | -4.393 | **1.12e-05 ***** |
| **iden_level-overall:gender** | **-0.6341122** | **0.1112390** | **-5.700** | **1.19e-08 ***** |
| **iden_level-specific:gender** | **-0.3771739** | **0.2967320** | **-1.271** | **0.20370** |
| **iden_level-overall:age** | **0.0002500** | **0.0015269** | **0.164** | **0.86997** |
| **iden_level-specific:age** | **0.0082476** | **0.0036735** | **2.245** | **0.02476 *** |
| **iden_level-overall:abundance(1)** | **-0.8711035** | **0.1195456** | **-7.287** | **3.17e-13 ***** |
| **iden_level-specific:abundance(1)** | **-1.7863417** | **0.3568523** | **-5.006** | **5.56e-07 ***** |
| **iden_level-overall:abundance(2)** | **-0.3154801** | **0.0715182** | **-4.411** | **1.03e-05 ***** |
| **iden_level-specific:abundance(2)** | **-0.5233203** | **0.2115954** | **-2.473** | **0.01339 *** |
| **iden_level0:gender:age** | **-0.0033282** | **0.0037232** | **-0.894** | **0.37137** |
| **iden_level1:gender:age** | **0.0154701** | **0.0085863** | **1.802** | **0.07159 .** |
| **iden_level2:gender:age** | **0.0227537** | **0.0107355** | **2.119** | **0.03405 *** |

Signif. codes: 0 ‘***’ 0.001 ‘**’ 0.01 ‘*’ 0.05 ‘.’ 0.1 ‘ ’ 1
