# Supplementary material for: Shifting Baselines on a Tropical Forest Frontier: Extirpations Drive Declines in Local Ecological Knowledge
Source: PLoS One. 2014 Jan 21;9(1):e86598. doi: 10.1371/journal.pone.0086598 (PMC3897741; doi:10.1371/journal.pone.0086598)
Supplement: Table S3 — Summary table of the best model for mammal identification. (DOC) [file pone.0086598.s003.doc]

**Table S3.** Summary table of the best model for mammal identification. The ability to identify a species at a particular level (overall: no id vs group-level or species-level, specific: group vs species levels) was modeled as a multinomial response in a surrogate Poisson (link=log) model. Thus the parameters of interest (highlighted in bold) are the interactions between id_level and the explanatory variables (respondent age (continuous), respondent gender (gender: male = 0, female = 1), species abundance (extant, extirpated), and their two-way interactions) [51]. Note that the main effects are marginal to the effects of interest and hence cannot be removed from the model. Village and individual respondent identity (ID) nested within village were treated as a random effects. We used AIC to select the most appropriate model and parameters were added and removed by hand in a stepwise manner. We considered all two way interactions between age, gender and species abundance (with identification level), but not three-way interactions. None of the two-way interactions were included in the best model. However, the models with two-way interactions were approximately equivalent (∆AIC = 0.8 – 4.4), but none of the coefficients for the interactive terms were significant (P>0.05. The model was somewhat under-dispersed (∑Peason's residuals^2 = 355, residual df = 669), indicating *p*-values are probably conservative.

| Generalized linear mixed model fit by maximum likelihood ['glmerMod'] | | | | |
| --- | --- | --- | --- | --- |
| Family: Poisson ( log ) | | | | |
| Formula: yes ~ iden_level + gender + age + abundance + (1 | village) + (1 | village/ID) + iden_level:gender + iden_level:age + iden_level:abundance  Data: mammals  AIC BIC logLik deviance  1731.1668 1798.9540 -850.5834 1701.1668  Random effects:  Groups Name Variance Std.Dev.  ID.hamlet (Intercept) 1.414e-12 1.189e-06  hamlet (Intercept) 2.313e-13 4.809e-07  hamlet.1 (Intercept) 3.961e-14 1.990e-07  Number of obs: 678, groups: ID:hamlet, 113; hamlet, 6 | | | | |
| Fixed effects | Estimate | Std. Error | z value | Pr(>|z|) |
| (Intercept) | 0.091146 | 0.126863 | 0.718 | 0.4725 |
| iden_level-overall | -0.398702 | 0.080445 | -4.956 | 7.19e-07 *** |
| iden_level-specific | -0.162105 | 0.169907 | -0.954 | 0.3400 |
| gender | -0.022595 | 0.061967 | -0.365 | 0.7154 |
| age | 0.001440 | 0.002856 | 0.504 | 0.6142 |
| abundance | -0.384685 | 0.067874 | -5.668 | 1.45e-08 *** |
| **iden_level-overall:gender** | **-0.062369** | **0.040174** | **-1.552** | **0.1206** |
| **iden_level-specific:gender** | **-0.002360** | **0.081718** | **-0.029** | **0.9770** |
| **iden_level-overall:age** | **0.002412** | **0.001850** | **1.304** | **0.1924** |
| **iden_level-specific:age** | **0.006713** | **0.003768** | **1.781** | **0.0749 .** |
| **iden_level-overall:abundance** | **-0.091844** | **0.038538** | **-2.383** | **0.0172 *** |
| **iden_level-specific:abundance** | **1.483987** | **0.096773** | **15.335** | **<2e-16 ***** |

Signif. codes: 0 ‘***’ 0.001 ‘**’ 0.01 ‘*’ 0.05 ‘.’ 0.1 ‘ ’ 1
